# Buffalo Medical and Surgical Journal

**Published:** 1877-03

**Authors:** 


### Editorial.

### Buffalo Medical and Surgical Journal.

After a period of repose,—apparent death, our JOURNAL faintly utters the dying words of Daniel Webster, "I still live," as much astonished at the fact as was the great statesman when he uttered the ever memorable words. Our JOURNAL, the pride and joy of our lives, could not withstand the financial depression of the recent Presidential Election, but we have agreed to unite our efforts and test the effects of transfusion, hoping it may yet revive and breathe again. The former editor under whose conduct the JOURNAL first sprung up and grew into popularity, has again consented to head the editorial staff, and with the aid of his associates and the Medical profession generally he now assures the readers of the JOURNAL that they will hereafter receive about the first of every month a Journal "what is a Journal," containing everything in Medicine and Surgery which is of any practical importance to know. As formerly it will be conducted wholly in the interest of the Medical Profession, and to it we look for patronage and support. Thus sustained it cannot fail or stop, but will be issued promptly and regularly, ample provision having now been made for the outlay of publication. Physicians in western New York are not willing to have it stop, and with the support which it has formerly received, it will not grow weary or tardy in appearance. It has for many years been a medium of communication with the profession, and it is now proposed to continue its influence. Progressive and inventive men are invited to support it, to send to it their best thoughts for publication, and to render it all the substantial aid in their power. It is to the profession we trustingly look as in former years for support. As Editors we promise earnest and impartial effort to make the JOURNAL a welcome visitor to every Physician's table.

Practical Medicine and Surgery is our specialty. We shall not take any other business even if it is offered. Medicine and Surgery in its widest, truest, and broadest scope is the sole object of our ambition. We shall study it carefully and practice it faithfully, and when better opportunity offers will tell those who read the JOURNAL what we have to say about it, and what the profession generally have to say about it.

You will all ask why the JOURNAL has been so late and irregular, and at last stopped altogether; frankly then, one young Physician, depending upon a small professional income could not incur the expense of publication, and wait for his subscribers to "help him out."

Hereafter, if we do not send you a readable, instructive, entertaining Journal you will not pay for it, but we expect by combined effort to send you the best small unpretending Medical and Surgical Journal published in the world. Now see if we redeem our promise, and do it so satisfactorily that the wherewith to continue publication will be urged upon us at every step.

